# Effect of Neoadjuvant Chemotherapy on Complications, in-Hospital Mortality, Length of Stay and Total Hospital Costs in Bladder Cancer Patients Undergoing Radical Cystectomy

**DOI:** 10.3390/cancers14051222

**Published:** 2022-02-26

**Authors:** Benedikt Hoeh, Rocco Simone Flammia, Lukas Hohenhorst, Gabriele Sorce, Francesco Chierigo, Andrea Panunzio, Zhe Tian, Fred Saad, Michele Gallucci, Alberto Briganti, Carlo Terrone, Shahrokh F. Shariat, Markus Graefen, Derya Tilki, Alessandro Antonelli, Luis A. Kluth, Philipp Mandel, Felix K. H. Chun, Pierre I. Karakiewicz

**Affiliations:** 1 Department of Urology, University Hospital Frankfurt, Goethe University Frankfurt am Main, 60596 Frankfurt am Main, Germany; luis.kluth@kgu.de (L.A.K.); philipp.mandel@kgu.de (P.M.); felix.chun@kgu.de (F.K.H.C.); 2 Cancer Prognostics and Health Outcomes Unit, Division of Urology, University of Montréal Health Center, Montréal, QC H4A 3J1, Canada; roccosimone92@gmail.com (R.S.F.); lukas@hohenhorst.com (L.H.); gab.sorce@googlemail.com (G.S.); francesco.chierigo@gmail.com (F.C.); panunzioandrea@virgilio.it (A.P.); zhe.tian24@gmail.com (Z.T.); fred.saad@umontreal.ca (F.S.); pierrekarakiewicz@gmail.com (P.I.K.); 3 Department of Maternal-Child and Urological Sciences, Sapienza Rome University, Policlinico Umberto I Hospital, 00185 Rome, Italy; michele.gallucci50@gmail.com; 4 Martini-Klinik Prostate Cancer Center, University Hospital Hamburg-Eppendorf, 20251 Hamburg, Germany; graefen@uke.de (M.G.); dtilki@me.com (D.T.); 5 Division of Experimental Oncology/Unit of Urology, URI, Urological Research Institute, IRCCS San Raffaele Scientific Institute, 20132 Milan, Italy; briganti.alberto@hsr.it; 6 Department of Surgical and Diagnostic Integrated Sciences (DISC), University of Genova, 16132 Genova, Italy; carlo.terrone@med.uniupo.it; 7 Department of Urology, University of Verona, Azienda Ospedaliera Universitaria Integrata di Verona, 37134 Verona, Italy; alessandro_antonelli@me.com; 8 Department of Urology, Comprehensive Cancer Center, Medical University of Vienna, 1090 Vienna, Austria; sfshariat@gmail.com; 9 Department of Urology, Weill Cornell Medical College, New York, NY 10021, USA; 10 Department of Urology, University of Texas Southwestern, Dallas, TX 75390, USA; 11 Department of Urology, Second Faculty of Medicine, Charles University, 128 08 Prague, Czech Republic; 12 Institute for Urology and Reproductive Health, I.M. Sechenov First Moscow State Medical University, 119992 Moscow, Russia; 13 Hourani Center for Applied Scientific Research, Al-Ahliyya Amman University, Amman 19328, Jordan; 14 Department of Urology, University Hospital Hamburg-Eppendorf, 20246 Hamburg, Germany; 15 Department of Urology, Koc University Hospital, Istanbul 34450, Turkey

**Keywords:** neoadjuvant chemotherapy, radical cystectomy, outcomes, bladder cancer, NIS, cost analysis, length of stay

## Abstract

**Simple Summary:**

Current guidelines recommend neoadjuvant chemotherapy (NAC) in muscle invasive, urothelial carcinoma of the urinary bladder patients treated with radical cystectomy (RC). However, large-scaled, contemporary data investigating the usage and effect of neoadjuvant chemotherapy prior to radical cystectomy on perioperative outcomes are scarce. We identified 4347 bladder cancer patients treated with RC between 2016 and 2019, relying on the National (Nationwide) Inpatient Sample (NIS) database. Of those, 805 (19%) received NAC. No differences for overall complication were recorded between RC patients treated with NAC vs. without. Specifically, NAC patients depicted lower rates of wound, cardiac, pulmonary and genitourinary complications. In line with this, in-hospital mortality rates as well as the length of stay were in favor for NAC patients. By contrast, NAC was associated with moderately higher total hospital costs. The current study recorded no detriment from NAC in the context of RC; however, the current study recorded persistently low rates of NAC contrary to current guidelines.

**Abstract:**

Background: To test for differences in complication rates, in-hospital mortality, length of stay (LOS) and total hospital costs (THCs) in patients treated with neoadjuvant chemotherapy (NAC) prior to radical cystectomy (RC). Methods: Within the National (Nationwide) Inpatient Sample (NIS) database (2016–2019), we identified RC-treated, non-metastatic, lymph-node negative bladder cancer patients, stratified by NAC status. Trend analyses, multivariable logistic, multivariable Poisson and multivariable linear regression models were used. Results: We identified 4347 RC-treated bladder cancer patients. Of those, 805 (19%) received NAC prior to RC. Overall, complications rates did not differ (65 vs. 66%; *p* = 0.7). However, NAC patients harbored lower rates of surgical site (6 vs. 9%), cardiac (13 vs. 19%) and genitourinary (5.5 vs. 9.7%) complications. In-hospital mortality (<1.7 vs. 1.8%) and LOS (6 vs. 7 days) was lower in NAC patients (all *p* < 0.05). Moreover, NAC was an independent predictor of shorter LOS in multivariable Poisson regression models (Risk ratio: 0.86; *p* < 0.001) and an independent predictor for higher THCs in multivariable linear regression models (Odds ratio: 1474$; *p* = 0.02). Conclusion: NAC was not associated with higher complication rates and in-hospital mortality. Contrary, NAC was associated with shorter LOS, yet moderately higher THCs. The current analysis suggests no detriment from NAC in the context of RC.

## 1. Introduction

Current guidelines recommend neoadjuvant chemotherapy (NAC) in muscle invasive, urothelial carcinoma of the urinary bladder (UCUB) patients treated with radical cystectomy (RC) [1,2,3,4,5,6,7,8]. Regardless of these recommendations, only two retrospective North American studies tested the effect of NAC on complications and mortality in a population-based fashion [9,10]. It is of note that both studies were of historical nature and one was additionally limited by its small-scale design (*n* = 878) [9]. Additionally, two larger-scale (*n* = 1340 and *n* = 1472)Scandinavian studies also tested the NAC effect on complication rates in a cohort of RC patients [11,12]. Irrespectively of their limitations related to sample size within the North American studies and related to patient origin within the Scandinavian data, no studies identified NAC as a risk factor for increased overall complication rates [9,11]. Despite the valuable contributions of these studies to the state of knowledge regarding NAC effect on perioperative outcomes, specific North American evidence concerning the NAC effect on perioperative outcomes is relatively weak, since it is derived from historical patient cohorts (2005–2011 and 2000–2009) [9,10]. Moreover, one study was limited by its sample size of RC patients (*n* = 878) among which only 78 (9%) patients received NAC in the setting of RC [9]. As a consequence, we tested the effect of NAC within a much larger and more contemporary North American cohort of RC patients, namely the National (Nationwide) Inpatient Sample (NIS) database between 2016 to 2019 [13]. The specific endpoints of interest consisted of complication rates, in-hospital mortality, length of stay (LOS) and total hospital costs (THCs). We hypothesized that NAC was not associated with less favorable complications and in-hospital mortality rates, as well as with higher LOS and higher THCs.

## 2. Material and Methods

### 2.1. Data Source

The NIS data repository represents a set of longitudinal hospital inpatient databases included in the Healthcare Cost and Utilization Project (HCUP), created by the Agency for Healthcare Research and Quality (AHRQ) through a federal state partnership [13]. The database includes approximately 20% of the United States’ inpatient hospitalizations. It incorporates patient and hospital information, including patients with Medicare, Medicaid, private insurance and other insurance types.

### 2.2. Study Population

Within the NIS database (2016–2019), we focused on patients with a primary diagnosis of bladder cancer (International Classification of Disease, Tenth Revision, Clinical Modification [ICD-10-CM] codes C67.0–C67.6, C67.8, C67.9) and without any prior cancer diagnosis (absence of ICD-10-CM code Z85.xx). Additionally, all patients included were aged >18 years and underwent RC, which was identified by ICD-10 Procedure Coding System [ICD-10-PCS] codes, as recently reported and validated by Lyon et al. [14]. Among those, patients with secondary ICD-10-CM codes indicating lymph-node invasion or metastatic stage were excluded from further analyses. NAC exposure status was defined according to ICD-10-CM code Z92.21. These inclusion criteria resulted in a final study cohort of 4347 eligible patients.

### 2.3. Outcomes of Interest

We focused on four outcomes: (1) in-hospital complication rates, (2) in-hospital mortality, (3) LOS and (4) THCs. Complication rates were defined using ICD-10-CM diagnostic codes, according to previously established methodology [15,16,17]. Overall, complication rates represented the sum of intraoperative and postoperative complications (bowel obstruction, transfusion, surgical site, cerebrovascular, gastro-intestinal, cardiac, pulmonary, genitourinary or other medical complications), as previously described [15,16,18,19]. Finally, total hospital charges, which are supplied by NIS, were converted to THCs using HCUP Cost-to-Charge ratios based on hospital accounting reports, in accordance with NIS methodological guidelines [13]. To facilitate comparison, all costs were additionally adjusted to 2016 dollars, relying on the overall Consumer Price Index [20].

### 2.4. Patient and Hospital Characteristics

Patient age, race/ethnicity (Caucasian, African American, Others), comorbidities and insurance status (Medicare, Medicaid, private insurance, other) were ascertained from the NIS. A modified Charlson Comorbidity Index (CCI) was used according to the Deyo adaptation for ICD-CM codes and patients were categorized as CCI 0–1 vs. ≥2, as previously published and validated relying on secondary ICD-10-CM codes [15,21,22]. Additional variables, which were ascertained from the NIS, consisted of hospital regions (northeast, midwest, south, west), income (1st, 2nd, 3rd, 4th income-quartile, unknown), hospital bed size (small, medium, large) and hospital teaching status (teaching vs. non-teaching). In order to gain teaching hospital status, institutions had to have either an American Medical Association-approved residency program, or needed to be a member of the Council of Teaching Hospitals, or had to have a ratio of 0.25 or higher of full-time equivalent interns and residents to non-nursing home beds [13].

### 2.5. Statistical Analyses

Descriptive statistics included frequencies and proportions for categorical variables. Means, medians, and interquartile ranges (IQR) were reported for continuously coded variables. The chi-square tested the statistical significance of proportions’ differences. The t-test and Kruskal–Wallis test examined the statistical significance of mean and distribution differences. Statistical analyses consisted of five steps. First, patient and RC-related characteristics, as well as outcome of interests were tabulated following stratification according to NAC status. Second, the estimated annual percentage change (EAPC) analyses were generated relying on the least squares linear regression methodology, as previously described [23,24]. EAPC analyses were weighted according to NIS methodology [13]. Third, separate sets of multivariable logistic regression models tested the effect of NAC on in-hospital complications, as well as on in-hospital mortality. Hereby, a forest plot was used to display the estimated odds ratio (OR) in multivariable logistic regression models for each specific complication. Fourth, since LOS is recorded as a count of days within the NIS, multivariable Poisson regression with log-link models were used to test the effect of neoadjuvant chemotherapy on LOS [15]. Fifth, multivariable linear regression models tested the effect of NAC on THCs. All multivariable models were performed after adjustment for clustering at the hospital level, using a Generalized Estimating Equation (GEE) function and were weighted accordingly to NIS methodology [13]. Covariables for adjustment consisted of age (per year), CCI (0–1 vs. ≥2), obesity (yes vs. no), gender, surgical approach (open vs. minimal-invasive), type of urinary diversion (incontinent vs. continent vs. unknown), year of surgery (per year), hospital bed size (small vs. medium vs. large), teaching status (yes vs. no), insurance status (Medicare, Medicaid, private insurance, other) and region (northeast, midwest, south, west). All tests were two sided with a level of significance set at *p* < 0.05 and the R software environment for statistical computing and graphics (version 3.4.3) was used for all analyses [25].

## 3. Results

### 3.1. Patients’ Characteristics and Temporal Trends

**Figure 1:**
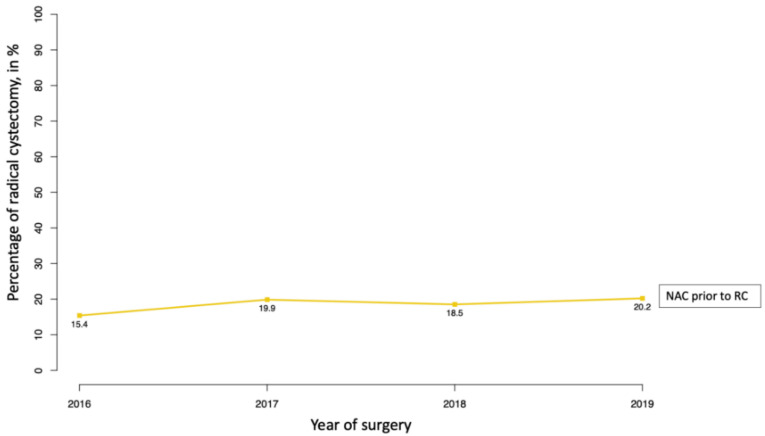
Annual rates of patients treated with neoadjuvant chemotherapy prior to radical cystectomy for non-metastatic bladder cancer within the National (Nationwide) Inpatient Sample database between 2016 and 2019. Abbreviations: EAPC = Estimated annual percentage change; 95%-CI = 95%-Confidence interval; RC = Radical cystectomy.

Between 2016 and 2019, 4347 RC patients were identified and eligible within the NIS (Table 1). Of those, 805 (19%) patients received NAC prior to RC. NAC patients were younger (67 vs. 70 years; *p* < 0.001), harbored higher proportions of CCI 0-1 (90 vs. 84%; *p* < 0.001) and were more often female (26 vs. 20%; *p* < 0.001). Moreover, in NAC patients, lymph-node dissection was performed more frequently (94 vs. 91%; *p* = 0.005) and a higher proportion of continent urinary diversion (7.5 vs. 5.1%; *p* = 0.007) was recorded. The annual NAC rates ranged from 15 to 20%, albeit without reaching statistical significance (EAPC: 7.1%; 95%-CI: −1.2–16.4%; *p* = 0.2; Figure 1).

### 3.2. Crude Rates of Outcomes of Interest

Overall complication rates were 63 vs. 65% in NAC vs. RC alone patients, respectively (*p* = 0.3). NAC patients exhibited lower rates of surgical site (6 vs. 9%; *p* < 0.001), cardiac (12 vs. 18%; *p* < 0.001), pulmonary (5 vs. 9%; *p* < 0.001) and genitourinary complications (5 vs. 9%; *p* = 0.002). No difference in complication rates was recorded in the residual complication groups (Table 2). In-hospital mortality rates were significantly lower in NAC patients (*p* = 0.02). Similarly, the median LOS was shorter in NAC patients (6 vs. 7 days; *p* < 0.001). Finally, no difference in median THCs was recorded (28,367 vs. 29,073$; *p* = 0.7).

### 3.3. Multivariable Analyses

**Figure 2:**
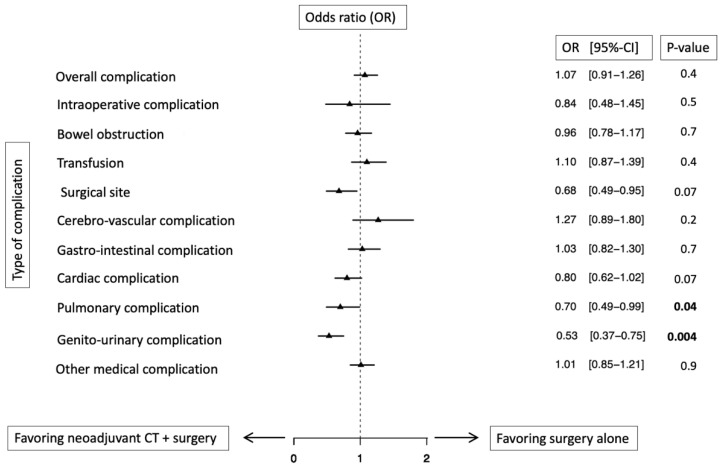
Forest plot displaying odds ratio of multivariable logistic regression analyses predicting in-hospital postoperative complications of urothelial carcinoma of the bladder patients treated with radical cystectomy, according to neoadjuvant chemotherapy usage (*ref.:* surgery without neoadjuvant chemotherapy), after adjustment for clustering. All models were weighted relying on a Generalized Estimation Equation (GEE) and were adjusted for clustering; Additional adjustment variables consisted of age, CCI, obesity, gender, surgical approach, type of urinary diversion, year of surgery, hospital bed size, teaching status, insurance status and region.; Abbreviation: OR: Odds ratio; 95% CI = 95% confidence interval.

In multivariable logistic regression models predicting overall complications, NAC failed to achieve statistical significance (Odds ratio [OR]: 1.07; 95%-CI: 0.91–1.26; *p* = 0.4). However, in separate multivariable logistic regression models addressing specific complication types (Figure 2), NAC was an independent predictor for lower pulmonary (OR: 0.70; 95%-CI: 0.49–0.99; *p* = 0.04) and genitourinary complications (OR: 0.53; 95%-CI: 0.37–0.75; *p* = 0.004). In multivariable logistic regression models predicting in-hospital mortality, NAC failed to achieve statistical significance (OR: 0.47; 95%-CI: 0.19–1.15; *p* = 0.1). Conversely, in multivariable Poisson regression models, NAC was an independent predictor for shorter LOS (Risk ratio: 0.86; 95%-CI: 0.83–0.90; *p* < 0.001). Finally, in multivariable linear regression models predicting THCs, NAC was an independent predictor for higher THCs (OR: 1858$; 95%-CI: 690–3026$; *p* = 0.02).

## 4. Discussion

The association between NAC and perioperative outcomes after RC in North American patients was only examined within two historical, population-based North American studies, of which one was limited by its sample size (*n* = 878) and low rates of NAC [9,10]. Consequently, we assessed the association between NAC prior to RC and four specific perioperative outcomes: (1) in-hospital complication rates, (2) in-hospitality mortality, (3) LOS and (4) THCs within the most contemporary data from within the NIS (2016–2019) and made several noteworthy findings.

First, we identified important differences in patient characteristics between NAC vs. non-NAC patients. Specifically, NAC patients were younger (67 vs. 70 years) and presented less frequent comorbidities (CCI 0-1: 90 vs. 84%). Conversely, in NAC patients, lymph-node dissection (94 vs. 91%) as well as continent urinary diversion (7.5 vs. 5.1%) were performed more frequently (Table 1). The current findings are in agreement with previously published studies, reporting that NAC patients tended to be younger and less morbid [9,11,12]. Consequently, a favorable selection bias may be associated with NAC and careful adjustment for those differences is clearly necessary.

Second, NAC rates increased from 15 to 20% in the current study years, albeit in a non-significant fashion (Figure 1). Despite the lack of statistical significance, the absolute rates suggest the broader use of NAC. Similar trends and differences between historical and contemporary years were previously reported and NAC rates ranged from 20 up to 32% NAC rates [9,26,27,28,29]. It is noteworthy that NAC rates within the current study are lower than in most previous reports. The reason for lower NAC rates in the current study may be manifold. For example, the histological subtype could not be restricted to UCUB only. As a consequence, unlike in previous studies, the denominator of RC patients in the current study included invariably and indisputably patients with histological subtypes that do not qualify for NAC. Moreover, the nature of participating institutions in the NIS vs. other epidemiological databases, such as the National Cancer database (NCDB) may contribute to this disparity [26,30,31]. Consequently, NAC rates in the current study, as well as in NIS in general cannot be compared directly to NAC rates in studies where histological subtypes UCUB is defined and where the denominator usually is restricted to UCUB. It is of note that the proportion of private insurance was significantly higher in NAC patients compared to RC only patients (37 vs. 26%). Whether this difference was unrelated or represents an example of healthcare access inequalities, can neither be confirmed nor rejected based on the current data.

Third, overall complication rates were 63 vs. 65% in, respectively NAC vs. RC patients (Table 2). These complications rates are highly comparable to complications rates reported in other, epidemiological studies addressing comparable outcomes of interest [9,11]. Overall, complication rates did not differ between NAC status (Figure 2). The current observations are in agreement with the North American study by Johnson et al., where 78 NAC patients were compared to 800 non-NAC patients undergoing RC between 2005 and 2011. Similarly, overall complication rates did not differ between NAC vs. non-NAC patients [9]. When the effect of NAC was investigated in an intermediate size Scandinavian cohort (*n* = 1340), NAC patients also exhibited either no differences or more favorable complication profiles than their non-NAC counterparts [11]. It is of interest that in the current study, lower specific complication rates for surgical site, cardiac and pulmonary events were recorded in NAC compared to RC patients (Table 2). Taken together, the current data regarding NAC complications relative to RC patients, in combination with the two other population-based studies, suggest no detriment from NAC when complication rates represent the outcome of interest. Consequently, clinicians should not restrict NAC referrals based on fear of higher rates of adverse outcomes if NAC is delivered. However, this assumption is based on the same selection criteria for NAC, as were applied to NAC patients within the current study, as well as to that of Johnson et al. (North American, *n* = 878) and Jerlström et al. (Scandinavian, *n* = 1340). Despite the combined evidence of the current study and the two other studies, the effect of NAC on postoperative complications should ideally be studied using prospective, large-scale data.

Fourth and finally, in the last part of the analysis, we tested the effect of NAC on LOS and on THCs. While LOS did not illustrate a disadvantage in NAC patients, NAC was associated with moderately higher THCs in multivariable linear regression models (OR: 1858$). As a consequence, it can be postulated that NAC does not predispose to longer LOS or substantially higher THCs (Table 3). Similar LOS observations were made by two other investigators. Contrary to the methodology applied in the current study, Jerlström et al., as well as Johnson et al., did not address these endpoints in multivariable models [9,11]. Consequently, the current findings examine the effect of NAC on LOS and THCs on the strictest methodological considerations.

The current study is not devoid of limitations and should be interpreted in the context of its retrospective and population-based design. Complications and mortality were limited to in-hospital rates. It is well known that complications and mortality rates may differ, when longer endpoints such as 30-, 60- or 90-day mortality rates are examined [32]. As a consequence, the current estimates represent the best-case scenario for all of the tested endpoints. Moreover, the current study cannot account for rates of delayed complications or readmissions at later time points. Furthermore, despite its very large sample size, the NIS has its intrinsic limitations. For example, histological subtype and detailed stages are not available. Consequently, NAC rates may have been diluted by inclusions of histological subtypes in which NAC is not as strongly recommended as for UCUB. Similarly, NAC rates may have been diluted by the inclusion of patients without muscle invasion, in whom an early cystectomy was performed. Moreover, perioperative data, such as operating time and intraoperative blood loss, are not provided by the NIS. Even though extensive adjustment for patient characteristics, which distinguished NAC patients from RC patients, such as age or CCI, was performed in all multivariable analyses, residual biases that were beyond the data provided by NIS, cannot be completely ruled out. Finally, NIS does not contain information about the specific type of NAC and the number of administered cycles. It is of note that this limitation accounts for other population-based studies [9].

## 5. Conclusions

NAC was not associated with higher complication rates and in-hospital mortality in a contemporary cohort of RC patients. By contrast NAC was associated with shorter LOS, yet moderately higher THCs. Taken together, this retrospective analysis suggests no detriment from NAC in the context of RC.

## Figures and Tables

**Table 1:** Descriptive characteristics of 4347 non-metastatic bladder cancer patients treated with radical cystectomy, stratified by neoadjuvant chemotherapy (CT) status, within the National (Nationwide) Inpatient Sample database between 2016 to 2019; All values are mean (Interquartile range) or frequencies (%).

	Overall, N = 4347	Surgery, N = 3542 (81%)	Neoadjuvant CT + Surgery, N = 805 (19%)	*p*-Value
**Age in years,** Median (IQR)	69 (62, 75)	70 (63, 76)	67 (61, 73)	<0.001
**Gender,** n (%)				<0.001
Female	936 (22%)	725 (20%)	211 (26%)	
Male	3411 (78%)	2817 (80%)	594 (74%)	
**Modified Charlson-Comorbidity Index,** n (%)				<0.001
0-1	3698 (85%)	2976 (84%)	722 (90%)	
≥2	649 (15%)	566 (16%)	83 (10%)	
**History of smoking,** n (%)	1656 (38%)	1292 (36%)	364 (45%)	<0.001
**Race/Ethnicity,** n (%)				0.6
Caucasian	3506 (81%)	2855 (81%)	651 (81%)	
African-American	256 (5.9%)	204 (5.8%)	52 (6.5%)	
Others	585 (13%)	483 (14%)	102 (13%)	
**Surgical approach,** n (%)				0.8
Open	2622 (60%)	2140 (60%)	482 (60%)	
Minimal-invasive	1725 (40%)	1402 (40%)	323 (40%)	
**Lymph-node dissection,** n (%)	3968 (91%)	3213 (91%)	755 (94%)	0.005
**Type of diversion,** n (%)				0.007
Incontinent	4078 (94%)	3335 (94%)	743 (92%)	
Continent	239 (5.3%)	179 (5%)	60 (7%)	
Other/Unkown	30 (0.7%)	*	<11 (<1.3%) *	
**Year of surgery,** n (%)				0.005
2016	1091 (25%)	923 (26%)	168 (21%)	
2017	1148 (26%)	920 (26%)	228 (28%)	
2018	1015 (23%)	827 (23%)	188 (23%)	
2019	1093 (25%)	872 (25%)	221 (27%)	
**Hospital bed size,** n (%)				0.14
Small	491 (11%)	397 (11%)	94 (12%)	
Medium	922 (21%)	772 (22%)	150 (19%)	
Large	2934 (67%)	2373 (67%)	561 (70%)	
**Teaching hospital,** n (%)	3911 (90%)	3174 (90%)	737 (92%)	0.1
**Region,** n (%)				0.6
Midwest	1176 (27%)	954 (27%)	222 (28%)	
Northeast	851 (20%)	706 (20%)	145 (18%)	
South	1530 (35%)	1257 (35%)	273 (34%)	
West	790 (18%)	625 (18%)	165 (20%)	
**Income,** n (%)				0.1
0-25-percentile	981 (23%)	815 (23%)	166 (21%)	
26-50-percentile	1153 (27%)	950 (27%)	203 (25%)	
51-75-percentile	1160 (27%)	923 (26%)	237 (29%)	
76-100-percentile	991 (23%)	800 (23%)	191 (24%)	
Other/Unknown	62 (1.4%)	*	<11 (<1.3%) *	
**Insurance provider,** n (%)				<0.001
Medicare	2717 (63%)	2284 (64%)	433 (54%)	
Private	1203 (28%)	908 (26%)	295 (37%)	
Medicaid	258 (5.9%)	210 (5.9%)	48 (6.0%)	
Others	169 (3.9%)	140 (4.0%)	29 (3.6%)	

* Cell counts of n <11 was not displayed according to Healthcare Cost and Utilization Project (HCUP) reporting guidelines.

**Table 2:** Complications rates, in-hospital mortality, length of stay and total hospital costs of 4347 non-metastatic carcinoma of the bladder patients treated with radical cystectomy, stratified by neoadjuvant chemotherapy status, within National (Nationwide) Inpatient Sample database between 2016 to 2019; All values are mean (Interquartile range) or frequencies (%).

	Overall, N = 4347	Surgery, N = 3542 (81%)	Neoadjuvant CT + Surgery, N = 805 (19%)	*p*-Value
**Overall complication** n (%)	2813 (65%)	2306 (65%)	507 (63%)	0.3
**Intraoperative** n (%)	100 (2.3%)	85 (2.4%)	15 (1.9%)	0.4
**Bowel obstruction** n (%)	914 (21%)	760 (21%)	154 (19%)	0.4
**Transfusion** n (%)	543 (12%)	439 (12%)	104 (13%)	0.7
**Surgical site** n (%)	379 (8.7%)	333 (9.4%)	46 (5.7%)	<0.001
**Cerebro-vascular** n (%)	248 (5.7%)	202 (5.7%)	46 (5.7%)	0.9
**Gastro-intestinal** n (%)	638 (15%)	523 (15%)	115 (14%)	0.7
**Cardiac** n (%)	743 (17%)	647 (18%)	96 (12%)	<0.001
**Pulmonary** n (%)	380 (8.7%)	337 (9.5%)	43 (5.3%)	<0.001
**Genito-urinary** n (%)	372 (8.6%)	332 (9.4%)	40 (5.0%)	0.002
**Other medical** n (%)	1650 (38%)	1363 (38%)	287 (36%)	0.1
**In-hospital mortality,** n (%)	66 (1.5%)	*	<11 (<1.4%)	0.02
**Length of stay,** Median (IQR)	6 (5, 9)	7 (5, 9)	6 (5, 8)	<0.001
**Total hospital costs in $^#^,** Median (IQR)	28,494 (21,639, 39,387)	28,367 (21,505, 39,397)	29,073 (22,178, 39,337)	0.2

^#^ Inflation adjusted (reference 2016-$), * Cell counts of n <11 was not displayed according to Healthcare Cost and Utilization Project (HCUP) reporting guidelines. Abbreviatons: IQR=Interquartile range; CT= Chemotherapy.

**Table 3:** Separate multivariable regression models predicting (a) overall complication, (b) in-hospital mortality, (c) length of stay and (d) total hospital costs in patients treated with radical cystectomy for malignancy of the urinary bladder National (Nationwide) Inpatient Sample database (2016–2019); All models were weighted relying on a Generalized Estimation Equation (GEE) function and were adjusted for hospital clustering; Additional adjustment variables consisted of age, CCI, obesity, gender, surgical approach, type of urinary diversion, year of surgery, hospital bed size, teaching status, insurance status and region.

Heading	Overall Complications ^1^			In-Hospital Mortality ^1^			Length of Stay ^2^			Total Hospital Costs ^3^		
Treatment Modality	**OR**	**95%-CI**	***p*** **-Value**	**OR**	**95%-CI**	***p*** **-Value**	**R** **isk Ratio**	**95%-CI**	***p*** **-Value**	**OR**	**95%-CI**	***p*** **-Value**
RC	Ref.			Ref.			Ref.			Ref.		
RC + neoadjuvant chemotherapy	1.07	0.91–1.26	0.4	0.47	0.19–1.15	0.1	0.86	0.83–0.90	<0.001	1858 ^4^	690–3026	**0.02**

^1^ Multivariable logistic regression model; ^2^ Multivariable Poisson regression model, additionally adjusted for overall complication; ^3^ Multivariable linear regression model, additionally adjusted for overall complication and length of stay; ^4^ Change in total hospital costs in $ per unit. Abbreviation: OR= Odds ratio; 95%-CI= 95%-confidence interval; RC= Radical cystectomy; Ref.: reference.

## Data Availability

All data generated for this study were from the National Inpatient Sample (NIS) database. The code for the analyses will be made available upon request.
